# Protective Effect of *Chrysanthemum boreale* Flower Extracts against A2E-Induced Retinal Damage in ARPE-19 Cell

**DOI:** 10.3390/antiox11040669

**Published:** 2022-03-30

**Authors:** Min Jung Kim, Dong Hee Kim, Han Sub Kwak, In-Sun Yu, Min Young Um

**Affiliations:** 1 Research Division of Food Functionality, Korea Food Research Institute, Wanju 55365, Korea; donghey543@naver.com (D.H.K.); dlstjs89@gmail.com (I.-S.Y.); myum@kfri.re.kr (M.Y.U.); 2 Research Division of Food Convergence, Korea Food Research Institute, Wanju 55365, Korea; hskwak@kfri.re.kr

**Keywords:** *Chrysanthemum boreale* Makino flower, A2E, age-related macular degeneration, A2E accumulation, A2E-induced cell death

## Abstract

In age-related macular degeneration, *N*-retinylidene-*N*-retinylethanolamine (A2E) accumulates in retinal pigment epithelium (RPE) cells and generates oxidative stress, which further induces cell death. Polyphenols are well known for their antioxidant and beneficial effects on vision. *Chrysanthemum boreale* Makino (CB) flowers, which contain flavonoids, have antioxidant activity. We hypothesized that polyphenols in ethanolic extracts of CB (CBE) and its fractions suppressed A2E-mediated ARPE-19 cell damage, a human RPE cell line. CBE is rich in polyphenols, shows antioxidant activity, and suppresses intracellular accumulation of A2E and cell death induced by A2E. Among the five fractions, the polyphenol content and antioxidant effect were in the order of the ethyl acetate fraction (EtOAc) > butanol fraction (BuOH) > hexane fraction (Hex) > dichloromethane fraction (CH_2_Cl_2_) > water fraction (H_2_O). In contrast, the inhibitory ability of A2E accumulation and A2E-induced cell death was highest in H_2_O, followed by BuOH. In the correlation analysis, polyphenols in the H_2_O and BuOH fractions had a significant positive correlation with antioxidant effects, but no significant correlation with cell damage caused by A2E. Our findings suggest that substances other than polyphenols present in CBE can suppress the effects of A2E, and further research is needed.

## 1. Introduction

Lipofuscin is a yellow-brown pigmented auto-fluorescent granule composed of lipids, oxidized proteins, and metals as a heterogeneous by-product of intracellular lysosomal degradation [1,2]. Oxidative stress, such as iron-catalyzed oxidative processes, induces lipofuscin formation and reactive oxygen species produced by damaged mitochondria accelerate lipofuscin accumulation [3]. Lipofuscin is an evidence of cellular senescence and aging because it accumulates continuously within the cell but is not degraded by the ubiquitin-proteasome system and cannot be removed via exocytosis [4]. Accumulation of lipofuscin occurs in various tissues, including the kidney, neurons, liver, heart, skeletal muscle, and retina, and each tissue has different characteristics and causes different diseases [5].

Lipofuscin in the retina is closely related to age-related macular degeneration (AMD), especially atrophy and geographic atrophy, which causes damage to the macula of the retina and central blindness in the elderly (>60 years old) [6]. According to the histopathological analysis of the diseased eye, the retinal pigment epithelium (RPE) is an important lesion site in AMD development and is the site of lipofuscin accumulation [7]. *N*-retinylidene-*N*-retinylethanolamine (A2E), a pyridinium bis-retinoid produced due to an abnormal vitamin A visual cycle, is a major component of RPE lipofuscin [4,8,9,10]. A2E in RPE cells is eliminated in youth and adults but is irreversible in those aged 50–60 years [11]. In vitro and in vivo, accumulated A2E can generate oxidative stress involving photoinducible free radicals and inhibit lysosomal function, cytochrome c oxygenase, and ATP-driven proton pump, further inducing inflammatory responses such as production and secretion of chemokines (IL-8, MCP-1, MCG, and MIP-1α) and cytokines (IL-1ß, IL-2, IL-6, and TNF-α), angiogenesis by enhancing the expression of vascular endothelial growth factor (VEGF), and RPE cell death, leading to retinal degeneration and blindness [9,12,13,14,15,16,17]. Therefore, studies on AMD prevention have focused on A2E-induced damage in RPE cells.

*Chrysanthemum boreale* Makino (CB) is a perennial plant belonging to the Asteraceae family that is distributed in Asia, including Korea, Japan, and China [18,19]. *Chrysanthemum* species are used as ingredients in food, cosmetics, and pharmaceuticals in many countries. The CB flower has been used as an oriental medicine to treat colitis, pneumonia, and stomatitis [20,21]. It has various biological inhibitory effects against cancer, inflammation, angiogenesis, hypertension, and bacteria [18,19,20,22,23]. CB flowers contain essential oils, polyacetylenes, and flavonoids. In particular, flavonoids present in many plant extracts are known to protect against oxidative stress in retinal diseases, including A2E [24]. Since CB also contains flavonoids, it may have a protective effect against cell damage caused by A2E, but this has not been studied yet.

Therefore, this study aimed to evaluate the protective effects of polyphenols present in the extracts of CB flowers (CBE) and its fractions on ARPE-19 cells, a human retinal pigment epithelial cell line against A2E-induced damage. The five fractions were analyzed for the total phenolic content (TPC) and total flavonoid content (TFC) of crude ethanolic CBE. The antioxidant and inhibitory effects of CBE and its five fractions on A2E accumulation and A2E-induced cell death in ARPE-19 cells were observed. The correlation between polyphenols present in CBE fractions and the inhibitory effect on A2E-induced cell damage was analyzed.

## 2. Materials and Methods

### 2.1. Reagents

The flower of CB was purchased from Jirisan Starmaru (Sancheong, Korea). The reagents used in this study were as follow: all-trans-retinal, triton X-100, ethanolamine, acetic acid, 2,2-diphenyl-1-picrylhydrazyl (DPPH), ascorbic acid, gallic acid, ABTS tablets, ABTS buffer, 2,4,6-tripyridyl-S-triazine (TPTZ), quercetin, potassium persulfate, iron(III) chloride (FeCl_3_), dimethyl sulfoxide (DMSO), chloroform, and Folin–Ciocalteu reagent from Sigma-Aldrich (St. Louis, MO, USA); sodium nitrite (NaNO_2_), aluminum (III) chloride (AlCl_3_), sodium hydroxide (NaOH), and sodium carbonate (Na_2_CO_3_) from Junsei Chemicals (Tokyo, Japan); water, methanol (MeOH), and acetonitrile from JT BAKER Chemical Co., (Phillipsburg, NJ, USA); ethanol (EtOH) from Merck (Darmstadt, Germany); 1 N hydrochloric acid from DAESUNG Co, Ltd. (Siheng, Korea); sodium acetate and trifluoroacetic acid (TFA) from Thermo Fisher Scientific (Waltham, MA, USA). The stock solution of lutein was 10 mM in dimethyl sulfoxide (DMSO). The final concentration of DMSO in all experiments was less than 0.1%. A2E and lutein levels were obtained from a previous study [25].

### 2.2. Ethanolic Extraction of CB and Fractionation of CBE

The dried CB flowers were powdered using a grinder and extracted by sonication in EtOH (10 g CB in 500 mL EtOH) using an ultrasonic processor (VCX 750; Sonics & Materials, Newtown, CT, USA) for 2 h at 4 °C. CBE was concentrated using a rotary evaporator (EYELA Co., Tokyo, Japan), lyophilized, and stored at −80 °C. The extraction yield (%) of CB was computed using:

Extraction yield (%) = ((mass of extract/mass of freeze-dried matter) ×100).

Five fractions were obtained via sequential extraction of CBE using hexane (Hex), dichloromethane (CH_2_Cl_2_), ethyl acetate (EtOAc), butanol (BuOH), and water (H_2_O). All fractions were concentrated using a rotary evaporator and stored at −80 °C. CBE and the five fractions were dissolved in DMSO to yield 100 mg/mL stock solutions. 

### 2.3. TPC and TFC

TPC was determined according to the modified Folin–Ciocalteu method [26]. The extract or fractions, diluted with MeOH, were mixed with 1 N Folin–Ciocalteu reagent for 5 min and then reacted with 700 mM sodium carbonate solution for 1 h. Then, the absorbance was measured at 765 nm using a spectrophotometer (SpectraMax M2e, Molecular Devices, Sunnyvale, CA, USA). A calibration curve for TPC quantification was constructed using gallic acid. The quantitative value was expressed as milligrams of gallic acid equivalent (GAE) per gram of dry weight of each sample (mg GAE/g dried sample).

TFCs were measured using the aluminum chloride colorimetric method [27]. The extract or fractions in MeOH were sequentially mixed with 5% NaNO_2_ for 6 min, with 10% AlCl_3_ for 5 min, and with 1 M NaOH for 15 min. The absorbance of the final reactant was measured at 492 nm using a spectrophotometer. A calibration curve for TFC quantification was constructed using quercetin. Quantitative values are expressed as milligrams of quercetin equivalent (QE) per gram of dry weight of each sample (mg QE/g dried sample).

### 2.4. Antioxidant Assay

DPPH radical-scavenging assay. The extract or fractions in MeOH were reacted with 0.4 mM DPPH solution for 30 min in the dark, and the absorbance of the reactant was measured at 517 nm. DPPH radical scavenging activity (%) was calculated as follows:

DPPH radical scavenging activity (%) = {(Ab_control_ − Ab_sample_)/Ab_control_} ×100.

ABTS radical-scavenging assay. ABTS radical cation (ABTS+•) was produced by mixing a 2.45 mM potassium persulfate solution and 7 mM ABTS solution (1:1) for 14–16 h in a darkroom and obtained by dilution in MeOH so that the absorbance value was 0.70 ± 0.02 at 734 nm. The extract or fractions were mixed with ABTS+• solution 1:1 and allowed to react in the dark for 10 min. The absorbance of the reaction solution was measured at 734 nm. ABTS radical scavenging activity (%) was calculated as follows:

ABTS radical scavenging activity (%) = {(Ab_control_ − Ab_sample_)/Ab_control_} ×100.

Ferric reducing antioxidant power (FRAP) assay. FRAP reagent was prepared by mixing acetate buffer (0.3 M in D.W., pH 3.6), TPTZ solution (10 mM in 40 mM HCl), and FeCl_3_ solution (20 mM in DW) at a ratio of 10:1:1 at 37 °C for 10 min. Then, the extract or fractions were mixed with FRAP reagent 1:10 and incubated at 37 °C for 10 min. The absorbance was measured at 593 nm. The quantitative value was obtained using the calibration curve with Trolox and expressed as mM of ferrous ions (Fe^2+^) per gram of sample (mM Fe^2+^/g sample). In all antioxidant assays, 30 μg/mL ascorbic acid was used as the positive control.

### 2.5. Cell Culture

ARPE-19 cells were purchased from American Type Culture Collection (ATCC; Manassas, VA, USA) and cultured in DMEM/F12 (Gibco, Gaithersburg, MD, USA) with 10% FBS (Gibco) and 1% penicillin/streptomycin (Gibco) at 37 °C in 5% CO_2_. When cells reached 70–80% confluence, they were dispensed into 96-well or 24-well plates (2 × 10^4^ cells/well or 1 × 10^5^ cells/well, respectively).

### 2.6. Cell Viability Assay

The cytotoxicity of CBE or its fractions was measured by the CCK-8 assay. ARPE-19 cells in 96-well plates were treated with CBE, fractions, or lutein at 5, 10, and 30 μg/mL for 24 h and incubated with CCK-8 reagent for 2 h. All controls were treated with 0.1% DMSO. The absorbance was measured at 450 nm using a spectrophotometer.

To determine the protective effect of CBE or fractions against A2E, ARPE-19 cells were sequentially treated with CBE, fractions, or lutein (5, 10, and 30 μg/mL) for 24 h, A2E (25 μM) for 24 h, and CCK-8 reagent for 2 h. The absorbance was estimated at 450 nm using a spectrophotometer and normalized as follows:

Cell viability = Ab_sample_/Ab_control_.

### 2.7. Measurement of Intracellular A2E Accumulation

ARPE-19 cells were incubated in 24-well plates (day 1), treated with CBE, fractions, or lutein (5, 10, and 30 μg/mL) for 24 h (days 2, 5, and 8), treated with A2E (10 μM) for 24 h (days 3, 6, and 9), and replaced with fresh complete media (days 4 and 7). Washing was performed with PBS between all the steps. Then, cells were harvested using 0.5% triton X-100 on the last day (day 10) and destroyed by sonication for 1 min. Cell lysates were used to measure the amount of protein using the BCA assay and the amount of A2E accumulated in the cells. A2E in the cell lysate was obtained in several steps: extraction with chloroform (three times), filtration with a polytetrafluoroethylene (PTFE) filter, evaporation with nitrogen gas, and dissolution in EtOH. The detection of A2E in EtOH was carried out using the Dionex Ultimate-3000 high-performance liquid chromatography (HPLC) system (Thermo Scientific, Sunnyvale, CA, USA), coupled with a quaternary pump, an autosampler, and a UV-Vis diode array detector (DAD3000; Thermo Scientific, Sunnyvale, CA, USA). For stationary phases, a reversed phase C18 column (4.6 × 250 mm; 5 μm particle size; Agilent Technologies, Santa Clara, CA, USA) was applied at a flow rates of 1.0 mL/min. The mobile phase consisted of water with 0.1% TFA (eluent A) and of acetonitrile with 0.1% TFA (eluent B). The gradient method was performed as follows: 85% eluent B (beginning), increased to 96% eluent B in 10 min, maintenance for 5 min, increase to 100% eluent B in 17 min, and maintenance for 25 min. A2E levels were quantified by measuring the amount of A2E using a standard curve and dividing it by the protein level.

### 2.8. Statistical Analysis

All experiments were performed at least in triplicate and all results are expressed as the mean ± standard deviation (SD). One-way analysis of variance (ANOVA) and Tukey’s honestly significant difference (HSD) tests were analyzed using GraphPad Prism 5 (GraphPad, San Diego, CA, USA). Using XLSTAT ver. 2017 (Addinsoft, Paris, France) the Pearson correlation coefficients were conducted to interpret the relationship between antioxidant activity and inhibition of A2E-induced damage and TPC/TFC.

## 3. Results

### 3.1. Extraction yield, TPC and TFC in Ethanolic CBE

The extraction yield, TPC, and TFC of CB are presented in Table 1. The yield of CBE was 14.3 ± 0.2%. The TPC and TFC in CBE were 64.08 ± 4.57 mg GAE/g and 51.51 ± 0.82 mg QE/g, respectively.

### 3.2. Antioxidant Effects of CBE

**Figure 1:**
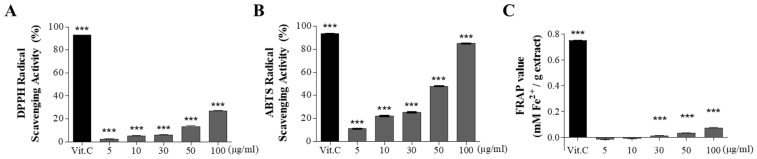
Antioxidant effects of CBE. (**A**) 2,2-Diphenyl-1-picrylhydrazyl (DPPH) radical scavenging assay. (**B**) 2,2′-Azino-bis(3-ethylbenzothiazoline-6-sulfonic acid) (ABTS) radical scavenging assay. (**C**) Ferric reducing antioxidant power (FRAP) assay. The values are the mean ± SD (n ≥ 4). *** *p* < 0.001 vs. blank group, one-way ANOVA with Tukey’s post hoc test.

The antioxidant activity of CBE was estimated using three different antioxidant assays, including DPPH, ABTS, and FRAP assays (Figure 1). DPPH radical scavenging activities of CBE were 2.44 ± 0.62, 5.23 ± 0.47, 6.10 ± 0.28, 13.33 ± 0.95, and 27.04 ± 0.52% at 5, 10, 30, 50, and 100 μg/mL concentrations, respectively. Scavenging activity of vitamin C (30 μg/mL) used as a positive control was 92.98 ± 0.10% of DPPH radical scavenging activity. ABTS radical scavenging activities of CBE were 11.13 ± 0.64, 22.29 ± 0.84, 25.39 ± 0.70, 48.00 ± 0.73, and 85.03 ± 0.46% at 5, 10, 30, 50, and 100 μg/mL concentrations, respectively, and that of vitamin C (30 μg/mL) was 93.39 ± 0.08%. The FRAP values of CBE were 0.014 ± 0.001, 0.035 ± 0.002, and 0.075 ± 0.005 mM Fe(II)/g CBE at 30, 50, and 100 μg/mL, respectively, and that of vitamin C was 0.749 ± 0.007. The antioxidant activity of CBE was significantly increased in a dose-dependent manner.

### 3.3. Inhibition of the Effects of A2E on ARPE-19 Cells by CBE

**Figure 2:**
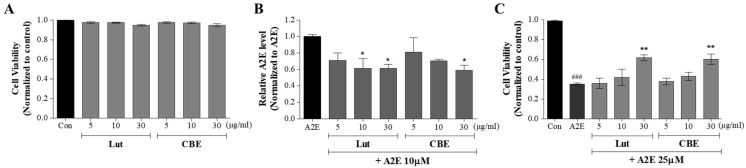
Inhibitory effects of CBE on A2E accumulation and A2E-induced cell death in ARPE-19 cells. (**A**) Nontoxicity of CBE to ARPE-19 cells. (**B**) Suppression of A2E accumulation in ARPE-19 cells by CBE. (**C**) Suppression of A2E-induced ARPE-19 cell death by CBE. The values represent the mean ± SD (*n* ≥ 3). ^###^ *p* < 0.001 vs. control group; * *p* < 0.05, ** *p* < 0.01 vs. A2E group, one-way ANOVA with Tukey’s post hoc test.

The cytotoxicity of CBE was estimated, and lutein was used as a positive control (Figure 2A). ARPE-19 cells were treated with CBE or lutein at 5, 10, and 30 μg/mL for 24 h, and then CCK-8 reagent was added to assess cell viability. Both CBE and lutein were not cytotoxic to ARPE-19 cells at any concentration. Next, to examine the effect of CBE on the intracellular accumulation of A2E, ARPE-19 cells were sequentially treated with CBE (5, 10, and 30 μg/mL) and A2E for 24 h each, and the A2E present in the cells was extracted and quantified using HPLC. Normalization was performed on A2E values obtained from non-CBE-treated ARPE-19 cells. As shown in Figure 2B, CBE inhibited A2E accumulation in a dose-dependent manner. Compared with the A2E-treated control, A2E accumulation was significantly reduced by 41.2% by CBE 30 μg/mL (* *p* < 0.05). Lutein also attenuated A2E accumulation at increasing concentrations. Significant differences were observed for lutein at 10 and 30 μg/mL, and lutein at 30 µg/mL did not show a significant difference from CBE at 30 µg/mL. 

As the accumulated high concentration of A2E can cause cell death, the suppressive effect of CBE on A2E-induced ARPE-19 cell death was monitored. A2E treatment (25 μM) significantly reduced cell viability by 64.7% (^###^ *p* < 0.001). In contrast, CBE treatment (30 μg/mL) significantly suppressed ARPE-19 cell death by A2E and showed a 1.7-fold increase in cell viability compared to cells treated with A2E alone. Lutein also significantly increased cell viability, like CBE.

### 3.4. The Yield of the Extraction, TPCs, and TFCs in Various Fractions

The extraction yield, TPC, and TFC of CB are presented in Table 2. The extraction yields of Hex, CH_2_Cl_2_, EtOAc, BuOH, and H_2_O fractions were 8.35 ± 0.2, 5.67 ± 0.2, 1.73 ± 0.2, 1.15 ± 0.1, and 5.49 ± 0.1%, respectively. TPC and TFC were present in the fraction with the identical pattern and were most abundant in the EtOAc fraction, followed by the BuOH, Hex, CH_2_Cl_2_, and H_2_O fractions in that order.

### 3.5. Antioxidant Effect of CBE Fractions

**Figure 3:**
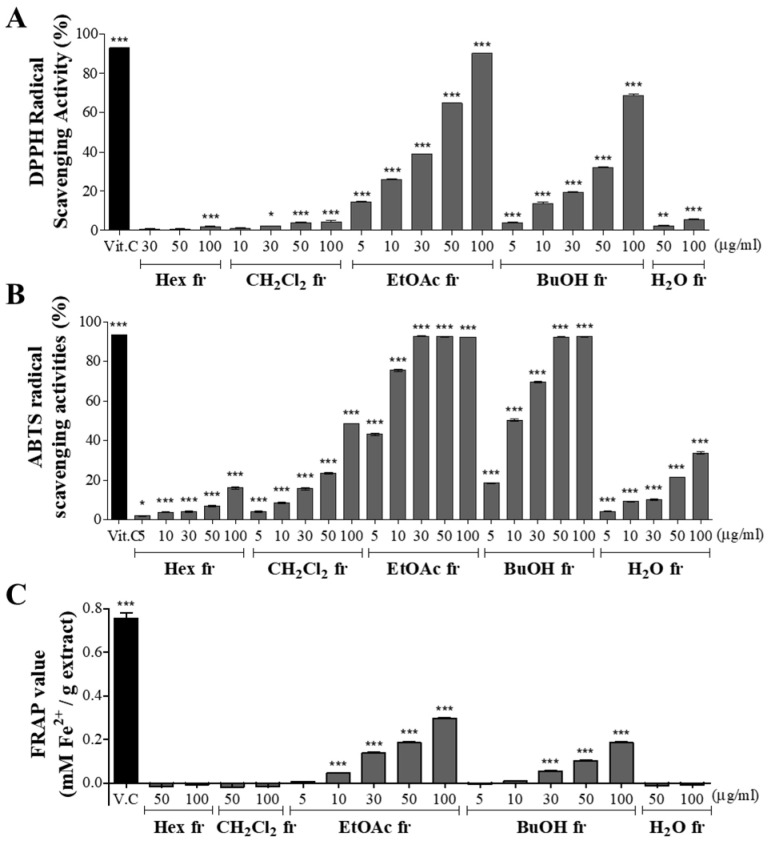
Antioxidant effects of five fractions. (**A**) DPPH radical scavenging assay. (**B**) ABTS radical scavenging assay. (**C**) FRAP assay. The values are the mean ± SD (*n* ≥ 3). * *p* < 0.05, ** *p* < 0.01, *** *p* < 0.001 vs. blank group, one-way ANOVA with Tukey’s post hoc test.

The antioxidant activities of the five CBE fractions were monitored using DPPH and ABTS radical-scavenging activity assays and FRAP. As shown in Figure 3, the DPPH radical scavenging activity of all fractions increased with increasing concentrations. The EtOAc fraction showed the highest radical-scavenging activity among the five fractions, followed by BuOH, CH_2_Cl_2_, H_2_O, and Hex.

A similar but stronger pattern of scavenging activity was observed in the ABTS assay. All CBE fractions showed a marked ABTS radical-scavenging activity in a dose-dependent manner. The highest ABTS radical scavenging activity was observed in the EtOAc fraction, followed by BuOH, CH_2_Cl_2_, H_2_O, and Hex, which was the same as the DPPH radical scavenging activity.

According to the FRAP assay, the quantified value was significantly increased in only two fractions, the EtOAc and BuOH fractions, in a concentration-dependent manner. In addition, the efficacy of the fractions in the FRAP assay was the weakest among the three antioxidant methods.

Overall, the antioxidant efficacy differed according to each antioxidant method. However, the trend was identical, in that the EtOAc fraction was the strongest among the five fractions, followed by the BuOH fraction. In particular, the EtOAc and BuOH fractions with high antioxidant activity contained the highest TPC and TFC among the fractions.

### 3.6. Inhibition of the Effects of A2E on ARPE-19 Cells by Some Fractions

**Figure 4:**
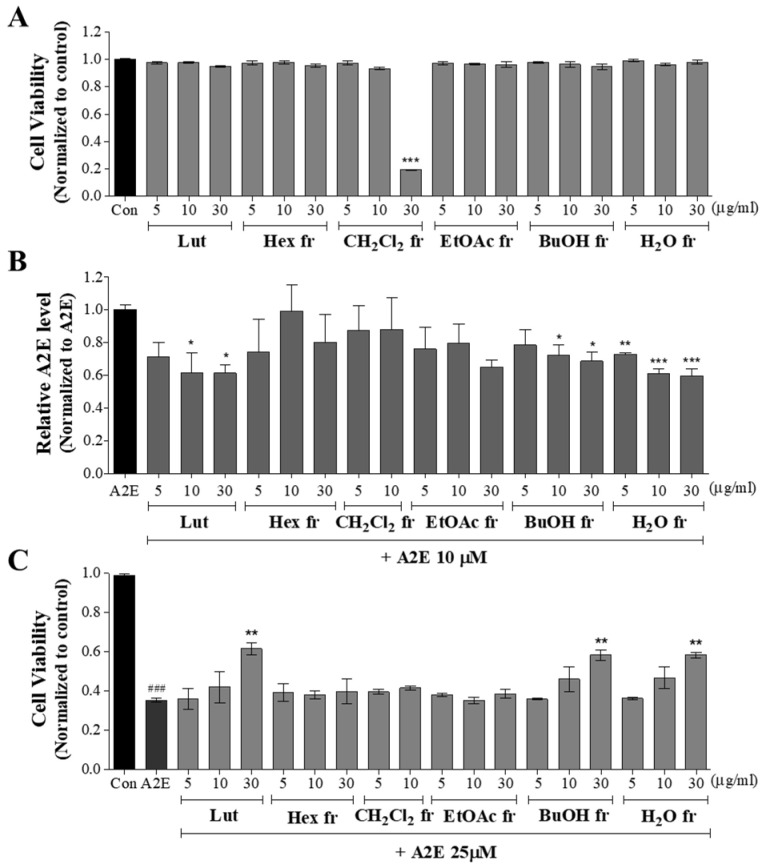
Suppressive effects of five fractions on A2E accumulation and A2E-induced cell death in ARPE-19 cells. (**A**) Cytotoxicity of five fractions to ARPE-19 cells. (**B**) Suppression of A2E accumulation in ARPE-19 cells by CBE. (**C**) Suppression of A2E-induced ARPE-19 cell death by CBE. The values represent the mean ± SD (*n* ≥ 3). ^###^ *p* < 0.001 vs. control group; * *p* < 0.05, ** *p* < 0.01, *** *p* < 0.001 vs. A2E group, one-way ANOVA with Tukey’s post hoc test.

The cytotoxicities of the five fractions were then evaluated. As shown in Figure 4A, most of the fractions in a concentration range from 5 to 30 μg/mL did not show cytotoxicity, except for the CH_2_Cl_2_ fraction at 30 μg/mL (*** *p* < 0.001). Therefore, the CH_2_Cl_2_ fraction at 30 μg/mL was excluded from subsequent experiments. Then, various concentration ranges of fractions were pretreated to ARPE-19 cells, and the suppressive effects of fractions on A2E accumulation in ARPE-19 cells were investigated (Figure 4B). The H_2_O, BuOH, and EtOAc fractions attenuated A2E accumulation with increasing concentrations. In particular, the H_2_O fraction was significant at all dose ranges. A2E accumulation was inhibited by 27.3, 39.1, and 40.5% at 5, 10, and 30 μg/mL concentrations in the H_2_O fraction, respectively. On the other hand, significant inhibition was observed in the BuOH fraction at 10 and 30 μg/mL (27.8 and 31.5% reduction) and in the EtOAc fraction at 30 μg/mL (35.1% reduction). Lutein also attenuated A2E accumulation in a concentration-dependent manner, and significance was observed at 10 and 30 μg/mL, which reduced 38.5 and 38.7%, respectively. Two other fractions, Hex and CH_2_Cl_2_, did not affect A2E accumulation in ARPE-19 cells.

The suppressive effects of the fractions on A2E-induced cell death in ARPE-19 cells were evaluated (Figure 4C). A2E (25 µM) decreased APRE-19 cell viability, and pretreatment with lutein effectively reduced the effect of A2E on ARPE-19 cells. Of the five fractions, only two, BuOH and H_2_O, were shown to inhibit A2E-induced cell death in a concentration-dependent manner. The experimental group pretreated with BuOH fraction and H_2_O fraction at 30 µg/mL showed a cell viability of 58.3% and 58.4%, which was 1.65 times higher than that of the A2E-treated control group. The cell viability obtained by pretreatment with the BuOH and H_2_O fractions at 30 µM was not significantly different from that of lutein at 30 µM.

### 3.7. Correlation Analysis between TPC/TFC and Antioxidant Activity/Inhibition of A2E-Induced Cell Damage

Correlation analyses were performed on only two fractions, showing a protective effect against A2E-induced damage. First, the correlation between the TPC/TFC ratio and antioxidant activity was analyzed (Table 3). In the BuOH fraction, TPC and TFC showed a significant positive correlation with DPPH (r = 0.991 and 0.993 for TPC and TFC, respectively) and FRAP (r = 0.993 and 0.993 for TPC and TFC, respectively) (*p* < 0.01). In the H_2_O fraction, TPC was associated with the scavenging activity against DPPH (r = 0.983) and ABTS (r = 0.990). Next, the correlation between these two fractions and A2E-induced damage was examined (Table 3). Both fractions showed a negative correlation with intracellular A2E levels and a positive correlation with the inhibition of cell death by A2E. However, no significant differences were observed in the correlation coefficients.

## 4. Discussion

This study demonstrated that: (1) CBE is rich in polyphenols, including phenolic compounds and flavonoids, and shows antioxidant activities in DPPH, ABTS, and FRAP assays; (2) CBE effectively inhibited A2E accumulation and A2E-induced cell death in ARPE-19 cells in a dose-dependent manner; (3) among the five fractions of CBE, EtOAc showed the highest polyphenol content and antioxidant activity, followed by BuOH; (4) however, A2E accumulation and A2E-induced cell death were suppressed the most in H_2_O, followed by BuOH; (5) according to the correlation analysis of BuOH and H_2_O fractions, TPC/TFC showed a significant positive correlation with antioxidant activity, but not with inhibitory activity against A2E-indued damage.

Oxidative stress is one of the most important factors in the pathogenesis and progression of ocular diseases such as retinal degeneration and dystrophy [28]. Under homeostatic conditions, living organisms strictly regulate reactive oxygen species (ROS) levels to support normal cellular functions and ensure redox signaling. However, when homeostasis is disrupted, and ROS accumulates, interactions between ROS and macromolecules result in oxidative stress, leading to cell damage and apoptosis. Aging is a major risk factor for the development of AMD due to ROS generation, promotion of oxidative stress accumulation, and decreased antioxidant defenses [14,15,29,30]. The RPE is susceptible to oxidative damage due to continuous light exposure, increased metabolic activity, and accumulation of oxidized lipoproteins [31]. ROS accelerate mitochondrial dysfunction, generation of lipoprotein and drusen, production of advanced glycation end products (AGEs), chronic inflammation, angiogenesis, and loss of antioxidant defenses over time, causing cell death in RPE cells in vitro and RPE cells from AMD patients [32,33,34]. Therefore, recent investigations have focused on reducing oxidative stress for AMD prevention.

Plant extracts have been studied to reduce oxidative stress and prevent AMD. The two modulators that regulate ROS levels in cells are enzymatic antioxidant molecules, including SOD, catalase, and glutathione metabolism-related enzymes, and non-enzymatic antioxidant molecules, including β-carotene, vitamins C and E, and glutathione [35]. Plant extracts are non-enzymatic substances. The large amounts of phenolic acids and flavonoids present in plant extracts exert antioxidant effects by removing radicals from the human body. In this study, CBE also contained phenolic compounds and flavonoids and was found to have antioxidant activity in DPPH, ABTS, and FRAP assays, consistent with previous studies.

Among the intrinsic factors present in the retina that induce oxidative stress, A2E is the main component [25]. Approximately 60–130 ng of A2E can be accumulated in 10^5^ human RPE cells [36,37]. Accumulated A2E in the retina is toxic when exposed to light such as blue light. Intracellular A2E generates ROS, accelerates chronic oxidative stress in RPE cells, and induces cell death leading to vision loss. Therefore, A2E has received widespread attention as a potential therapeutic target for AMD prevention. A2E does not trigger DNA damage at low concentrations (~20 μM) but causes cell death when it continuously accumulates in cells at concentrations of approximately 25 and 50 µM [36,37,38,39]. On the other hand, photo-oxidation and photodegradation of A2E occur even under ambient lighting including visible light, which is a general experimental environment [40]. A2E can generate single oxygen (^1^O_2_) and superoxide anion (O_2_^•−^) after photo-oxidation, and can act as a substrate by double bonding with ^1^O_2_ and radical oxygen species in RPE cells. A2E (20 μM) by itself or in the presence of visible light (630 nm) does not affect ATP production in mitochondria in RPE cells, but generates H_2_O_2_ and O_2_^•−^ and reduces mitochondrial and cytoplasmic SOD activities and catalase activity, which are implicated in antioxidant mechanisms [41]. A2E also releases dicarbonyl methylglyoxal (MG) and glyoxal (GO), which cause cell damage after photolysis, and forms proteins that react with MG. That is, ROS, oxidized A2E, and photolysis products can induce cytotoxicity in RPE cells. As described above, the antioxidant activity of CBE was confirmed by DPPH, ABTS, and FRAP assays. The effect of plant extracts on A2E-induced cytotoxicity is determined by examining whether they inhibit A2E accumulation in cells or A2E-induced cell death. CBE successfully suppressed the accumulation of A2E in ARPE-19 cells and A2E-laden ARPE-19 cell death in a dose-dependent manner, similar to lutein. Thus, polyphenol-rich CBE had not only antioxidant effects but also had suppressive effects on A2E-induced cell damage. This is the first study to report that CBE protects ARPE-19 cells against A2E, but several studies have demonstrated that plant extracts containing high amounts of polyphenols showed protective effects against A2E or other oxidative stress inducers. *Arctium lappa* L. leaves rich in polyphenols had antioxidant activity and decreased intracellular A2E accumulation, A2E-laden cell death in vitro, and light-induced retinal damage in vivo [25]. Polyphenol-rich *Vaccinium uliginosum* L. and *Prunella vulgaris* var. L. extract also prevented photooxidative damage and apoptosis caused by A2E in vitro and in vivo [42,43,44].

The five fractions of CBE also contained TPC and TFC and had antioxidant activity but with different degrees of efficacy. TPC and TFC were abundant in the EtOAc > BuOH > Hex > CH_2_Cl_2_ > H_2_O fractions. As the solubility and polarity of the extracted compounds are different, the extraction yield, TPC, and TFC in various solvents are different [45]. The antioxidant efficacy of the fractions was highest in EtOAc and BuOH fractions, which tended to be similar to those of TPC and TFC. However, the inhibition efficacy of A2E-induced damage was not significantly correlated with TPC and TFC present in the fractions. The H_2_O fraction with the lowest TPC, no TFC, and low antioxidant activity showed the best inhibition of cell damage caused by A2E. Considering the substances produced by A2E, this may be due to the oxidized A2E, MG-protein adducts, AGEs, receptor for advanced glycation end products (RAGE), and AGE-modified proteins, except for ROS. This hypothesis can be supported by previous report [46]. Among four kinds of polyphenols (quercetin, cyanidin-3-glucoside, ferulic acid, and chlorogenic acid), all decrease intracellular ROS level but only two polyphenols (quercetin and cyanidin-3-glucoside) activated cell viability. These two polyphenols commonly reduced photo-oxidation of A2E, formation of oxidized-A2E, MG-protein adduct, and RAGE mRNA expression. Polyphenols present in the H_2_O fraction of CBE may also have similar effects to quercetin and cyanidin-3-glucoside. Some polyphenols, including linarin, acacetin 7-O-b-d-glucopyranosyl-(1→2)[α-L-rhamnopyranosyl-(1→6)]-β-d-glucopyranoside, chlorogenic acid, 3,5-di-O-caffeoylquinic acid, and apigenin, were identified in CBE [47,48]. In addition, CB flowers contain chrysanthemin, sesquiterpenoids, and essential oil [49]. Chrysanthemin is an anthocyanin and a water-soluble pigment. Bilberries and blueberries containing chrysanthemin are known to have protective effects against A2E. Anthocyanins from bilberries protect RPE cells against A2E photo-oxidation, and bilberry extract suppresses RPE cell damage from visible light and ROS generation [50,51]. Anthocyanins from blueberries prevented A2E-induced cytotoxicity by reducing A2E-epoxidation. Some volatile compounds, including camphor, borneol, α-pinene, camphene, 1,8-cineole, germacrene-D, α-thujene, α-muurolene, piperitol, and hinesol, were identified in the essential oils of the CB flowers [52,53]. The essential oil of CB flowers is usually extracted by steam distillation, which vaporizes water and is obtained by redissolving it in an organic solvent. Although essential oils are mainly soluble in organic solvents, they sometimes remain in the aqueous phase. There are few studies on compositional analysis of phytochemicals present in CBE. Therefore, quercetin or cyanidin-3-glucoside may be present in H_2_O fraction of CBE. Quercetin is detected in the H_2_O fraction from methanolic extract of Syzygium cumini (L.) seed [54]. Cyanidin-3-glucoside is a type of anthocyanin and is water soluble [55]. However, since trace amount of polyphenols were detected in H_2_O fraction, there is a possibility that non-polyphenols also have a protective effect against A2E. Although follow-up studies are needed to identify polyphenol and non-polyphenol components that have cytoprotective effects against A2E in H_2_O fraction of CBE, CBE itself has been shown to inhibit A2E-induced cell damage. As A2E is a biomarker in AMD patients, CBE could be a medicinal plant with protective effects against AMD.

## 5. Conclusions

Overall, the results suggest that CB can be used as a functional plant to attenuate AMD development and progression. CBE, which contains a large amount of phenolic acid and flavonoids, has antioxidant effects and effectively inhibits A2E accumulation and A2E-induced cell death. In the CBE fraction experiment, the EtOAc fraction, containing a large amount of polyphenol, also had high antioxidant activity, but no cytoprotective effect against A2E was found. The protective effect against A2E was most potent in the H_2_O fraction, in which no flavonoids were detected. Finally, we propose that the non-polyphenolic components present in CBE may effectively prevent AMD.

## Figures and Tables

**Table 1:** The extraction yields, total phenolic content (TPC), and total flavonoids content (TFC) in crude ethanolic CB extract.

Analyzed Materials		Extraction Yield (%, *w*/*w*)	TPC (mg GAE/g)	TFC (mg QE/g)
Crude	CB	14.3 ± 0.2 ^(1)^	64.08 ± 4.57	51.51 ± 0.82

GAE, gallic acid equivalents; QE, quercetin equivalent. ^(1)^ Values are represented as mean ± SD (*n* = 4).

**Table 2:** The extraction yields, TPCs, and TFCs in five fractions.

Analyzed Materials		Extraction Yield (%, *w*/*w*)	TPCs (mg GAE/g)	TFCs (mg QE/g)
Fraction	Hex	8.35 ±0.2 ^(1)^	31.88 ± 8.58	52.31 ± 2.56
	CH_2_Cl_2_	5.67 ± 0.2	18.07 ± 4.36	3.85 ± 0.57
	EtOAc	1.73 ± 0.2	199.27 ± 0.38	155.76 ± 1.00
	BuOH	1.15 ± 0.1	112.36 ± 0.86	70.8 ± 1.33
	H_2_O	5.49 ± 0.1	5.38 ± 1.54	-

Hex, hexane; CH_2_Cl_2_, dichloromethane; EtOAc, ethyl acetate; BuOH, butanol; H_2_O, water. ^(1)^ Values are represented as mean ± SD (*n* = 4).

**Table 3:** Pearson correlation coefficients for TPC, TFC, antioxidant activity, and inhibition of A2E accumulation and A2E-induced cell death of the BuOH and H_2_O fractions.

Pearson Correlation Coefficient (r)						
Analyzed Materials		DPPH	ABTS	FRAP	A2E Accumulation	Inhibition of A2E-Induce Cell Death
BuOH	TPC	0.991 **	0.829	0.993 **	−0.912	0.974
	TFC	0.993 **	0.802	0.993 **	−0.874	0.951
H_2_O ^(1)^	TPC	0.983 *	0.990 **	- ^(2)^	−0.822	0.988

** Correlation is significant at the 0.01 level. * Correlation is significant at the 0.05 level. ^(1)^ Since TFC was not detected in the H_2_O fraction, correlation analysis was performed only for TPC. ^(2)^ Correlation analysis was not performed because scavenging activity against FRAP was not shown.

## Data Availability

Data is contained within the article.
